# APE1 Activity is Controlled by Non‐G‐Quadruplex Conformations in Single‐ and Double‐Stranded G‐Quadruplex Constructs

**DOI:** 10.1002/chem.202503023

**Published:** 2025-12-17

**Authors:** Brianna L. Trabucco, Aaron M. Fleming, Cynthia J. Burrows

**Affiliations:** ^1^ Department of Chemistry University of Utah Salt Lake City USA

**Keywords:** Abasic sites, APE1, DNA damage, endonucleases, G‐quadruplexes

## Abstract

Apurinic/apyrimidinic endonuclease‐1 (APE1) is a repair enzyme that efficiently cleaves abasic (AP) site damage in duplex DNA. Reports of *in vitro* activity assays between APE1 and single‐stranded G‐quadruplex (ssG4) reveal significant decreases in the endonuclease activity. Here, we identify that the low yields observed represent cleavage of the noncanonical folds that did not adopt a complete G4 fold. This conclusion is supported through circular dichroism analysis and activity assays analyzing the cleavage rate, folding impact on cleavage, and product inhibition. Studies were performed on AP‐containing ssG4 and duplex‐embedded G4 scaffolds. The CD spectra of a non‐G4 containing potential quadruplex sequence reveal a noncanonical structure. APE1 can cleave an AP in these non‐G4 conformation(s) in high yields comparable to the preferred duplex substrate. There is direct evidence of decreasing APE1 activity with increasing G4 folding in ssG4 and duplex‐G‐quadruplex‐duplex (DGD) systems. Also observed is a positional dependency on yield in the non‐G4 DGD scaffolds, but not in the non‐G4 ssG4 scaffolds. In conclusion, our studies provide evidence that APE1 efficiently cleaves noncanonical conformations in G4‐like structures, highlighting the control of secondary structure on APE1 endonuclease activity.

## Graphical abstract

G‐quadruplex (G4) folding in DNA is inhibitory to AP endonuclease, although the protein still binds well to G4s. Curiously, partially folded G‐rich sequences, possibly transient hairpins, containing AP are very good substrates for cleavage by APE1. Herein, the enzyme activity and G4 folding are studied in single‐stranded vs. duplex contexts.

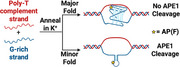

## 1 Introduction

DNA repair pathways rely on enzymes to recognize and process damaged sites within the genome that may exist in diverse structural motifs. Among the key enzymes involved, human apurinic/apyrimidinic endonuclease‐1 (APE1) is a multifunctional protein that is critical to the base excision repair (BER) pathway as it efficiently cleaves abasic sites (AP) in duplex DNA. Failure to repair such lesions can compromise genome integrity, ultimately leading to mutations found in cancer and neurodegenerative diseases [1, 2, 3, 4]. In addition to canonical substrates, APE1 encounters DNA lesions in nonduplex DNA structures, which can have varying impacts on binding and endonuclease activity; such structures include single‐stranded DNA, bulges, primer‐template junctions, H‐DNA, and G‐quadruplex (G4) folds [5, 6, 7, 8, 9, 10, 11, 12]. Scaffolds with G4 motifs are identified as particularly inhibitory, with the endonuclease activity of APE1 significantly reduced when an AP is within this noncanonical structure [8, 10, 13]. In the present study, we demonstrate that when an APE1 substrate resides in a duplex–G‐quadruplex–duplex (DGD) scaffold that mimics the genomic context of a G4, the enzymatic cleavage is inversely dependent on the extent of G4 folding, although APE1 still binds to folded G4s.

**FIGURE 1:**
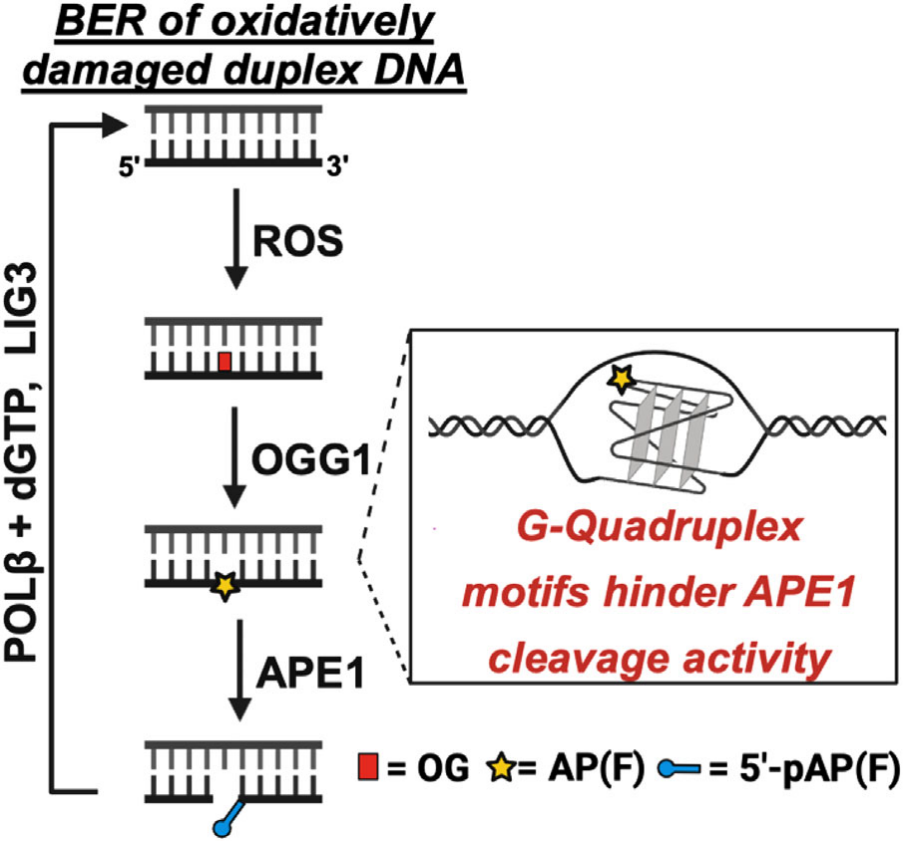
Short‐patch BER pathway initiated by oxidative damage.

Oxidative damage to DNA is a route to the formation of AP substrates for APE1 cleavage. The most sensitive site to oxidation is at 2′‐deoxyguanosine (dG) nucleotides to yield 8‐oxo‐7,8‐dihydro‐2′‐deoxyguanosine (dOG), which is enriched in G‐rich genomic regions such as those that are potential G4‐forming sequences (PQS) [14, 15, 16]. In the human genome, PQSs are enriched at gene promoters and telomeres, in which the damage in promoters has the potential to impact transcription [17, 18, 19, 20, 21, 22]. The product dOG is preferentially a short‐patch BER substrate that begins with the removal of the damaged base via the glycosylase OGG1 to yield an AP site that destabilizes the duplex [15, 23, 24]. The AP site is recognized by APE1 that binds the destabilized site and kinks the duplex for extrusion of the AP site in the active site to catalyze hydrolysis of the 5’ phosphodiester bond yielding a 3’ hydroxyl and a 5’‐deoxyribosephosphate (5’‐dRP) [25]. In the present studies, the chemically labile AP site is replaced with the chemically stable tetrahydrofuran (F) mimic that is a comparable substrate for APE1 [26]. Herein, the nomenclature used is F for the AP substrate, and the 5’‐dRP product is termed 5’‐pF. In the cellular context, after APE1 cleavage of the AP, polymerase beta (POLβ) first removes the 5’‐dRP by a lyase reaction to generate a gap, and then the enzyme will act in its polymerase role to fill the gap using dGTP opposite dC in the templating strand. Finally, the strand is sealed by DNA ligase III (LIG3) to return the DNA to its native state to avoid mutations (Figure 1) [14, 15, 27].

Sequencing studies by our group and others identified G‐rich gene promoter sequences as hotspots for oxidative damage and dOG formation [16, 28, 29, 30]. We also found using synthetically‐defined reporter plasmids in mammalian cell cultures a direct correlation between oxidative damage in G4 promoter regions (*VEGF* and *NEIL3*) and upregulation of these genes [9, 15, 17]. Activity and binding studies by our group found that APE1 will bind G4 folds with similar affinity to that of a duplex containing an AP or F site, but the endonuclease activity is inhibited [8]. Interestingly, this APE1‐G4 binding interaction may act as a hub for recruiting transcription factors for gene expression [17, 31]. ChIP‐Seq analyses supports a mechanism in which oxidative damage in a promoter PQS results in a binding interaction between APE1 and the folded G4 prior to gene induction [22]. Other laboratories have further validated different aspects of this mechanism in various gene promoters and involving a variety of transcription factors [18, 19, 20, 21]. While much is now known about the transcription‐modulating interaction between APE1 and AP‐bearing G4 folds, the endonuclease reaction for the G4 folds embedded between duplex handles, similar to the genomic context, remains incompletely understood.

*In vitro* studies investigating the inhibitory impact of G4 scaffolds on APE1 endonuclease activity have employed single‐stranded substrates due to the relative ease of promoting G4 folding [8, 10, 11, 13, 32, 33, 34]. These systems typically consist of short sequences with at least four G‐tracks, each containing three or more dG nucleotides [15]. However, attempts to anneal these G‐rich sequences with their exact, or “true,” complement favors duplex formation over G4 folding, as duplex is the thermodynamically preferred structure [8]. To overcome this challenge, a scaffold consisting of two duplex handles with a G4 embedded in between them, referred to as a DGD motif, is utilized in these studies. Supported by cryo‐EM structural studies, the annealing of a *c‐MYC* promoter PQS strand with a poly‐dT‐containing complement led to a G4 fold with duplex handles on each end and an extruded poly‐dT strand opposite the G4 [35]. Inspired by these studies, we adapted this scaffold to the *VEGF* promoter sequence, a well‐known G4‐forming PQS associated with dOG formation, BER, and gene regulation, which has been studied and characterized in *vitro* by our laboratory and others [8, 13, 15, 17, 36]. Previously, we found by CD and NMR that the *VEGF* DGD could form a G4 motif, including when an F is present in loop positions [37]. The present studies explore the endonuclease interaction between APE1 and DGD scaffolds, which better model the double‐stranded G4 context found in the genome. We now hypothesize that additional structures are present in G4‐based annealing conditions, and the low cleavage yields observed in the presence of APE1 are due to F‐modified non‐G4 structure(s). Our results highlight that as the folded G4 presence in a DGD context increases, the endonuclease‐catalyzed cleavage yield decreases, and it is also dependent on the location of the AP(F) site. Overall, DNA secondary structures are active modulators of APE1 repair enzyme activity, offering insight relevant to both enzyme function and overall genome maintenance.

## 2 Results

**FIGURE 2:**
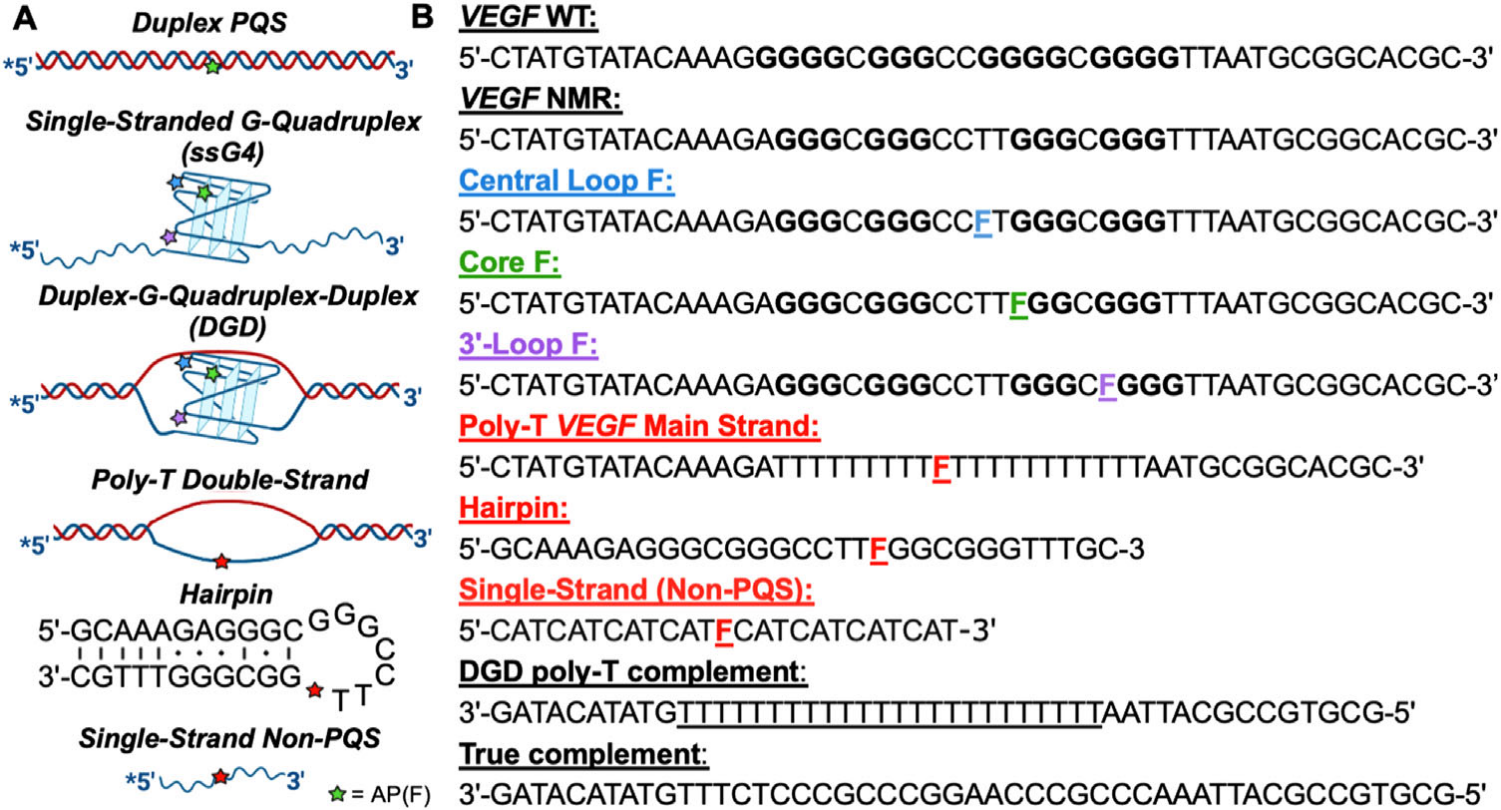
(A) 5’‐^32^P radiolabeled AP(F)‐modified DNA motifs. (B) Sequences of WT and modified *VEGF* strands, complements, and control strands (red), which include the poly‐T *VEGF* main strand, hairpin, and single‐stranded non‐PQS strands. The *VEGF* NMR sequence was adapted from the WT sequence to remove additional G nucleotides to favor a single type of fold [38]. The *VEGF* NMR sequence was individually AP(F)‐modified in a central loop, core, or 3’‐loop positions. The *VEGF* NMR strand was annealed to DGD poly‐dT complement for DGD scaffolds and annealed to the true complement strand for duplex contexts. The poly‐T *VEGF* main strand was annealed to DGD poly‐dT complement for the poly‐T double‐strand scaffold, representing a double‐stranded non‐G4 scaffold. The ssG4 and ss‐non‐PQS strands have no complement.

*Comparison of Folding across VEGF PQS Scaffolds‐* In these studies, we compared *VEGF* DGD constructs with *VEGF* duplex DNA, *VEGF* single‐stranded G4s, and a non‐PQS single‐strand oligomer across a variety of studies (Figure 2A). The *VEGF* sequence studied within, termed *VEGF* NMR, was mutated from *VEGF* WT for prior NMR analysis to allow a single G4 fold to exist whose structure has been previously reported by Yang and coworkers (Figure 2B) [38]. Being interested in the impact of F in the G4 fold, we placed F sites in the central loop (blue), a core position (green), and the 3’‐loop (purple) of the PQS (Figure 2B).

Folding studies were performed and analyzed by CD to characterize the G4 folding abilities across the DGD and ssG4 scaffolds when introducing the F sites at one of three different locations. All scaffolds were treated by thermodynamic folding, which began with the introduction of 100 mM of either K^+^ ions, which promote G4 formation, or Li^+^ ions, which do not promote G4 formation but will allow duplex formation, followed by heating to 90°C and slowly cooling to room temperature [39]. For DGD formation, this step was followed by annealing of the poly‐dT‐containing complement to the G‐rich strand for 24 h at 4°C to allow the formation of the duplex handles following a literature protocol [35]. For duplex substrates, the true complement was added to the core F strand.

**FIGURE 3:**
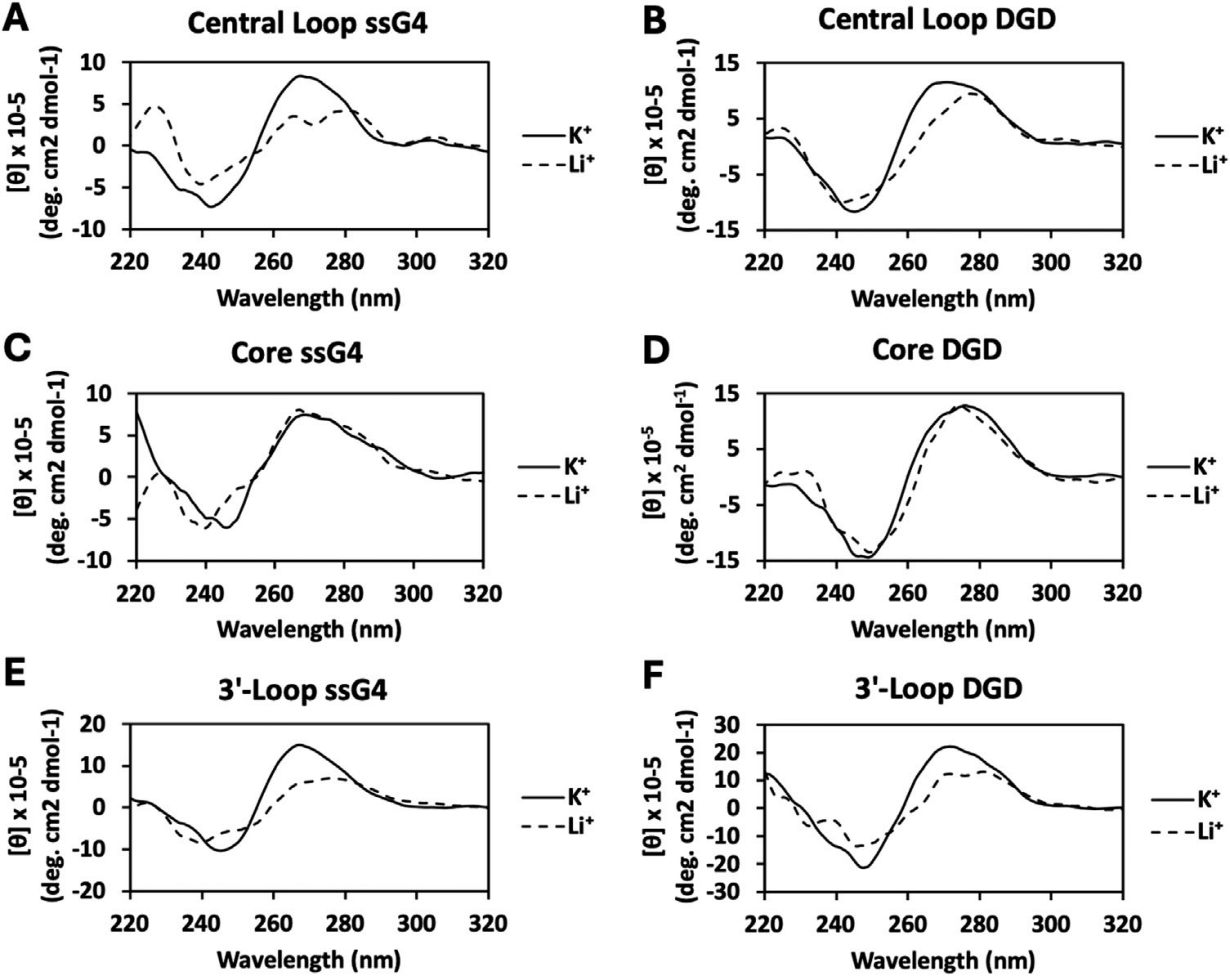
CD analysis for thermodynamically folded F‐modified *VEGF* ssG4 (A, C, E) and DGD (B, D, F) motifs folded in 100 mM Li^+^ (dashed) or K^+^ ion (solid) solutions.

Previous DGD CD studies by our group found a shift in maxima between the ssG4 scaffold, with a 263 maximum and 245 minimum, and the DGD scaffold, with a 271 nm maximum and 245 minimum, both indicating the presence of a parallel‐stranded G4 motif [37]. The shift in wavelength of the maximum peak for the DGD scaffold is attributed to the presence of the poly‐dT complement strand and the duplex handles. CD analysis of the canonical duplex shows a maximum at 275 nm (Figure S1), a longer wavelength than that of the ssG4, further supporting the upward shift in wavelength in the duplex‐containing DGD spectra. This trend is exhibited in the CD data for unmodified *VEGF* NMR ssG4 and DGD scaffolds (Figure S1). Both *VEGF* NMR G4 scaffolds showed different behavior in the presence of K^+^ vs. Li^+^ (Figure S1), which could be used to monitor the presence of a G4 [37]. The CD spectrum was also obtained for the single‐strand non‐PQS substrate, which differed from that of the *VEGF* NMR ssG4 (Figure S1). The CD data in this study for the central loop and 3’‐loop F modifications show a shift from a shorter wavelength maximum in the ssG4 scaffolds to a longer wavelength in the DGD scaffolds, suggesting the presence of parallel‐stranded G4 motifs (Figures 3A, B, E, F). Between the minimum and maximum values and the difference of spectra between the K^+^ and Li^+^ studies, it can be confirmed that there is a G4 motif present for both loop F‐modified ssG4 and DGD scaffolds. On the other hand, when F is present in a core position for both a ssG4 or DGD scaffold, the minima and maxima do not match the signature values, and the K^+^ and Li^+^ spectra overlap, indicative of no G4 structure being present (Figures 3C, D).

The structure and size differences of each scaffold were further investigated by native polyacrylamide gel electrophoresis (PAGE) (Figure S2). In addition to the previously mentioned scaffolds, a modified poly‐T *VEGF* was introduced, which replaces the PQS portion of the *VEGF* NMR WT with a poly‐T sequence (Figure 2). When annealed with the DGD poly‐T complement, this scaffold should represent a non‐G4 DGD with an unstructured motif embedded between duplex handles. Both the single‐stranded poly‐T *VEGF* and double‐stranded system with the poly‐T complement were included. CD was performed on the double strand and confirmed the presence of the duplex handles (Figure S3). As expected, the duplex, ssG4 (or single‐stranded poly‐T *VEGF*), and DGD (or double‐stranded poly‐T *VEGF*) scaffolds all migrated to different locations on the gel, indicating different sizes and structures. It can be confirmed that the DGD has a complement present, as ssG4 travelled further than the DGD on the gel due to its smaller size and higher flexibility. It can also be confirmed that duplex and DGD hold different structures because they are approximately the same size but are seen in different locations on the gel. These results hold consistently for each location of F modification in the scaffolds. The poly‐T *VEGF* single strand was aligned with the ssG4 scaffolds, and poly‐T *VEGF* double strand aligned with the DGD scaffolds. Additionally, the core and loop ssG4 scaffolds show another band below the ssG4, most prominent in the central loop ssG4, which could be indicative of an alternative fold of the strand, such as a hairpin. These CD and native PAGE analyses confirm that the duplex, ssG4, and DGD scaffolds are their own unique scaffolds while also highlighting that placing an F in a loop position allows G4 folding in these ssG4 and DGD scaffolds, but placing it in a core position inhibits G4 folding.

*Time Course Activity Assays of DGD Cleavage by APE1‐* Activity assays with APE1 were performed on all F modification locations in both ssG4 and DGD scaffolds over a 15‐minute time course. Duplex and single‐stranded non‐PQS structures, which cannot form a G4, were studied as structural references. Reactions were performed by adding 100 nM APE1 to an annealed solution containing approximately 100 nM of 5’‐^32^P radiolabeled DNA (1 G‐rich strand:1.2 complement), 20 mM Tris (pH 7.4 at 30°C), 100 mM KOAc, 1 mM Mg(OAc)_2_, and 1 mM DTT and then stopped at time point intervals from 30 s to 15 min. Reactions were analyzed by denaturing PAGE, and yields were calculated from the band intensity of the 5’‐^32^P radiolabeled reactant and product from the audioradiogram. Aligning with previous ssG4 data from our laboratory, we found that [Mg^2+^] does not impact the cleavage reactions (Figure S4) [8].

**FIGURE 4:**
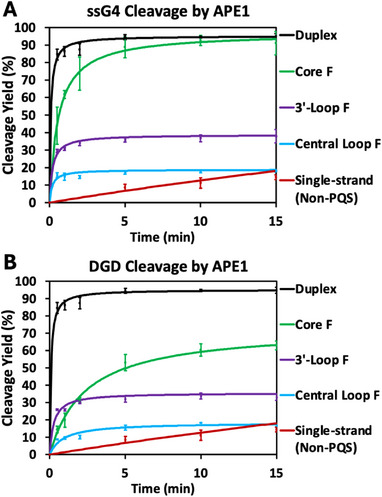
Time course APE1 activity assays with F in the secondary structures studied. (A) The F substrates were in ssG4 contexts, and (B) in DGD motifs. The control structures, duplex, and single‐stranded non‐PQS are also provided in both panels. The reactions were quenched at 30 s, 1 min, 2 min, 5 min, 10 min, and 15 min. Reactions were performed in solutions of approximately 100 nM DNA (5’‐^32^P radiolabeled), 100 nM WT APE1, 20 mM Tris (pH 7.4), 100 mM KOAc, 1 mM DTT, and 1 mM Mg(OAc)_2_. Each graph presents three different locations of the F lesion in the ssG4 and DGD motifs, including central loop, core, and 3’‐loop positions, as shown in Figure 2. The same time courses for the duplex and single‐stranded non‐PQS are presented in both graphs.

**FIGURE 5:**
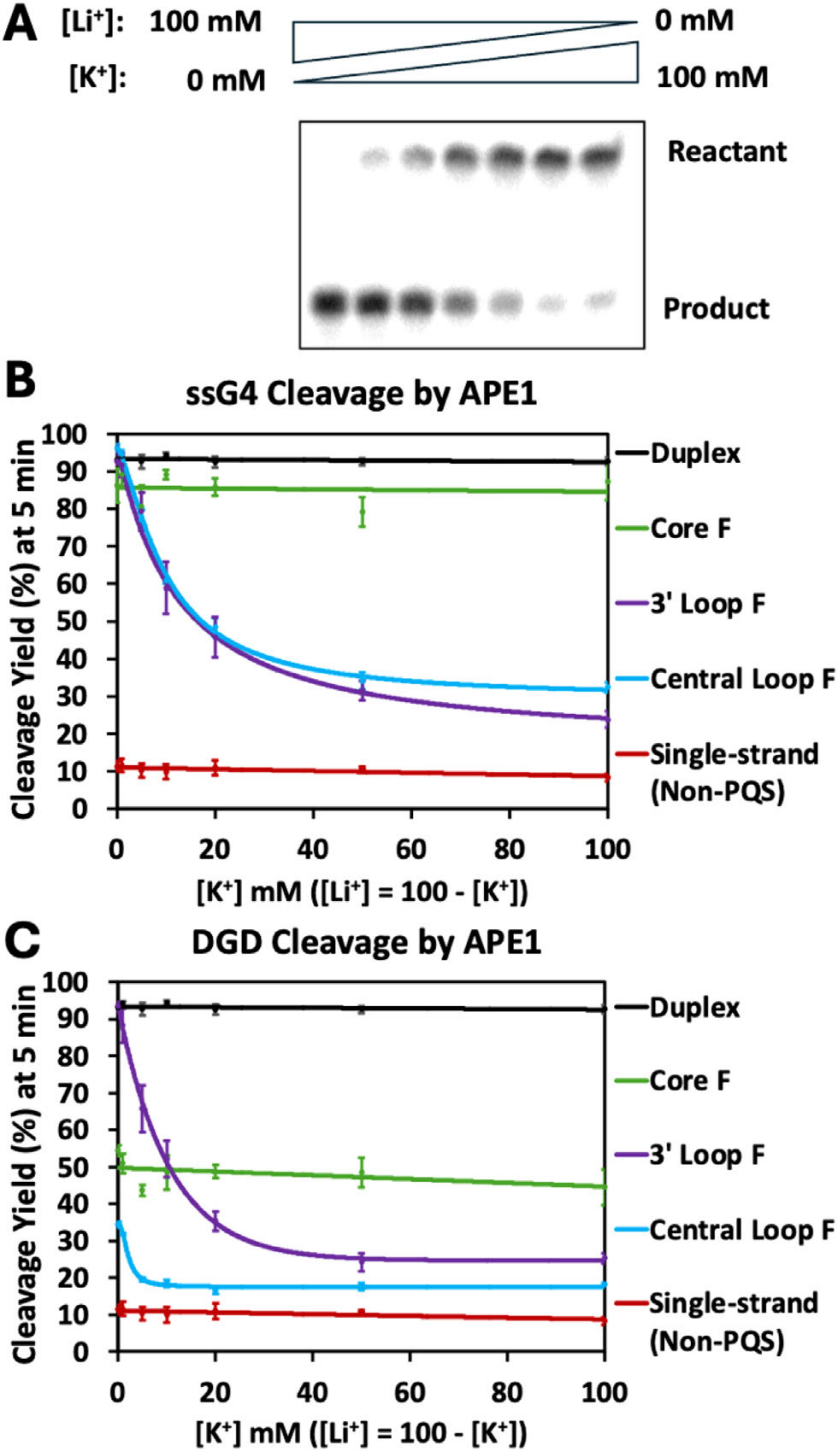
K^+^/Li^+^ ion‐dependent APE1 activity assays performed on ssG4 and DGD scaffolds at around a 1:1 ratio of APE1:DNA (5’‐^32^P radiolabeled + nonradiolabeled). Each reaction buffer contained LiOAc and/or KOAc at an ionic strength of 100 mM. The reactions were quenched after allowing them to react for 5 min. (A) Denaturing PAGE gel of the ion concentration‐dependent APE1 cleavage of 3’‐loop DGD as the K^+^ ion presence increased. Central loop, core, and 3’‐loop F modified (B), ssG4, and (C) DGD cleavage by APE1 over a K^+^/Li^+^ ion‐dependent gradient with duplex and single‐strand controls.

The duplex time course for endonuclease cleavage was, as expected, quickly reaching a maximum yield of ∼90% (Figures 5A and 5B, black trace). The ss‐non‐PQS strand had undetectable cleavage by APE1 at time points under 5 min, and there then emerged a slow increase of cleavage from 9% at 5 min to the maximum yield of approximately 75% at 3 h (Figures 5A, 5B and S5, red trace) [40, 41]. Compared to the duplex substrate, the loop F‐modified ssG4 and DGD scaffolds, which were shown to adopt G4 motifs by CD, displayed inhibited activity, reaching near maximum yields by ∼5 min, the time point used for subsequent studies (Figures 4A and 5B). At 5 min, the 3’‐loop F DGD exhibited an average yield of 32% (Figure 4B, purple trace) and the ssG4 an average yield of 36% (Figure 4A, purple trace). These yields and the general shapes of the time curves for the DGD and ssG4 were closely aligned, in the sense that cleavage occurred rapidly but plateaued after 2–3 min.

In the central loop F scaffolds, at 5 min, the DGD was cleaved with an average yield of 16% (Figure 4B, blue trace), and the ssG4 was cleaved with a 17% average yield (Figure 4A, blue trace), which again align closely. These values are lower than those of the 3’‐loop F scaffolds, indicating a positional dependency of the F modification on cleavage yields by APE1.

When the F is placed in the core position, which does not allow G4 formation, the ssG4 and DGD‐based scaffolds show significantly different yields. The core F‐modified DGD is cleaved at 5 min at a 53% average yield (Figure 4B, green trace), and surprisingly, the ssG4 experiences average yields near that of the duplex within error (Figure 4A, green trace). It can also be observed that the core F DGD is slower to reach its maximum cleavage yield compared to the loop F DGD scaffolds. Overall, these data identify that ssG4 and DGD scaffolds that adopt G4 folds produce lower F cleavage yields by APE1 compared to the core F substrates that fail to fully adopt G4 motifs or the classical duplex substrate. The most surprising observations are that non‐G4, but G‐rich, structures containing an F modification are quite good substrates for APE1, suggesting that they may have a structure partially resembling a duplex. A time‐course analysis was also performed on the F‐modified poly‐T double‐strand system, which lacks a G‐rich region and lacks structure between the duplex handles (Figure S6). This data showed a similarly shaped curve to that of the core F DGD as well as a similar yield of 60% at 5 min. This highlights that APE1 cleavage of non‐G4 scaffolds does not require G‐rich strands. In addition to the poly‐T double‐strand non‐G4 control, an F‐modified hairpin control was designed and utilized (Figure 2). Interestingly, this hairpin shows a time‐course curve that nearly overlays with the poly‐T double‐strand curve with a yield of 66% at 5 min (Figure S6). Again, this aligns well with the core F DGD data. Therefore, APE1 cleaves non‐G4 structures embedded between duplexes, with or without a G‐rich region, and hairpins in a similar manner, which is more efficient than cleavage of G4 structures.

*K^+^/Li^+^ Impact on DGD Cleavage by APE1‐* APE1 activity assays were performed with a K^+^/Li^+^ concentration gradient, with varying ratios of K^+^ ions to Li^+^ ions while maintaining the ionic strength at 100 mM monovalent cation(s). Annealing in the salt mixtures was performed in the same way as previous K^+^‐only reactions. In previous studies, most scaffolds reach near‐complete cleavage by the 5‐min point, except for the single‐strand non‐PQS; therefore, 5‐min time points were utilized in this salt gradient study. The purpose of these assays was to study the impact of G4 presence on APE1 cleavage activity, as K^+^ supports G4 formation and Li^+^ does not [37, 38, 39]. Therefore, in the beginning of the gradient in which there was only Li^+^ present, there should not be G4s present, but upon adding a small amount of K^+^, G4 folding occurs to some extent. An example of a denaturing PAGE gel from this reaction gradient is depicted in Figure 5A, specifically showing the 3’‐loop F modified DGD scaffold. The x‐axis on these graphs (Figures 5B and 5C) shows [K^+^], in which the total [K^+^] + [Li^+^] adds to 100 mM. As depicted in both Figures 5B and 5C, duplex, ss‐non‐PQS, and core F ssG4 and DGD scaffolds, which are the scaffolds that cannot form a G4 motif, show no APE1 cleavage dependency on the presence of K^+^ or Li^+^ ions and align well with previously reported yields from the time course studies (Figures 4A and 4B). The core F ssG4 shows a cleavage yield near that of the duplex substrate at around 85% (Figure 5B), while the core F DGD shows lower yields averaging 47% (Figure 5C). On the other hand, both loop F modified ssG4 and DGD scaffolds show decreasing yield with increasing [K^+^] and therefore increasing presence of G4 motifs in solution (Figures 5B, 5C). As G4 motif formation is complete with increasing [K^+^], the previously observed yields are replicated (Figures 4A and 4B).

In the ssG4 context with only Li^+^ ions present, and therefore no G4 motif, both loop F modifications reach > 90% yield, similarly to that observed for the duplex DNA substrate (Figure 5B, purple and blue). This is roughly ninefold higher cleavage than APE1 can perform on the single‐strand non‐PQS, which is cleaved at an average of about 10% (Figures 5B and 5C, red traces). This also highlights that in Li^+^, there is no significant F positional dependency for APE1 cleavage in ssG4 scaffolds.

In the DGD scaffolds there was F positional dependency when the APE1 cleavage reactions were conducted in a solution containing only Li^+^ ions. Here, the 3’‐loop F DGD (Figure 5C, purple trace), the F modification location closest to the 3’‐duplex handle, showed a similar curve to that of the ssG4 (Figure 5B, purple trace), where they both reach yields of >90%. The core F DGD once again shows no K^+^/Li^+^ dependency when being cleaved by APE1, producing an average cleavage yield of 47% (Figure 5C, green trace), which is nearly twofold lower than the ssG4 context with an 85% yield (Figure 5B, green trace). Finally, the central loop DGD was cleaved in Li^+^ at 34% yield (Figure 5C, blue trace), which is nearly threefold lower than that of its ssG4, which had a >90% average yield (Figure 5B, blue trace).

In summary, these K^+^/Li^+^ activity assays show a consistent trend of APE1 cleavage activity decreasing as stable G4 motifs are formed by the addition of more K^+^ ions. These data also depict a positional dependency in DGD scaffolds in Li^+^, which lack a G4 motif, with increasing yield as the F modification is positioned closer to the 3’‐duplex handle (Figure 5C). This positional dependency is not observed in the ssG4 scaffolds and demonstrates that ssG4 and DGD scaffolds behave differently when an F is present for cleavage by APE1. Thus, the context in which an F‐containing G4 resides impacts the cleavage yields.

**FIGURE 6:**
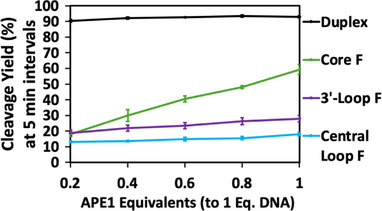
Cleavage activity assays for APE1 titration additions in duplex and F‐modified DGD scaffolds. Approximately 0.2 eq. of APE1 was added to the reaction solution every 5 min, and an aliquot was removed and quenched at each 5‐min point for analysis. The final addition of APE1 was quenched at 25 min with an approximate 1:1 equivalency of [APE1] and [DNA].

*APE1 Product Inhibition with DGD Scaffolds‐* Product inhibition studies were performed on the DGD scaffolds with APE1 to explore the cause of the low cleavage yields in the presence of G4 motifs. Reactions were performed with approximately 100 nM DNA samples (5’‐^32^P radiolabeled) by adding approximately 0.2 equivalents of APE1 (20 nM), allowing to react for 5 min, removing an aliquot, stopping the reaction, adding another 0.2 equivalents to the main reaction tube, and continuing these steps until there was 1 equivalent of APE1 to DNA. By the time the final reaction was stopped, APE1 had been present in solution for 25 min, giving ample time for product turnover between additions. As depicted in Figure 6, the duplex substrate reaches its maximum yield of ∼90% within 5 min of adding the first 0.2 equivalents of APE1. Very minimal increases in yield over all 5 additions of APE1 were observed for both loop F DGD scaffolds (Figure 6, purple and blue traces). Both showed near maximal cleavage yields upon the first addition of APE1 when compared to the maximum yields observed in Figure 5. There was a significant increase in yield with each addition of APE1 to the core F DGD, which cannot fold into a 3‐tetrad G4 (Figure 6, green trace). Previously observed yields (Figures 4B and 5C) for the core DGD were not obtained until 1 equivalent of APE1 to DNA was present upon the final addition.

**FIGURE 7:**
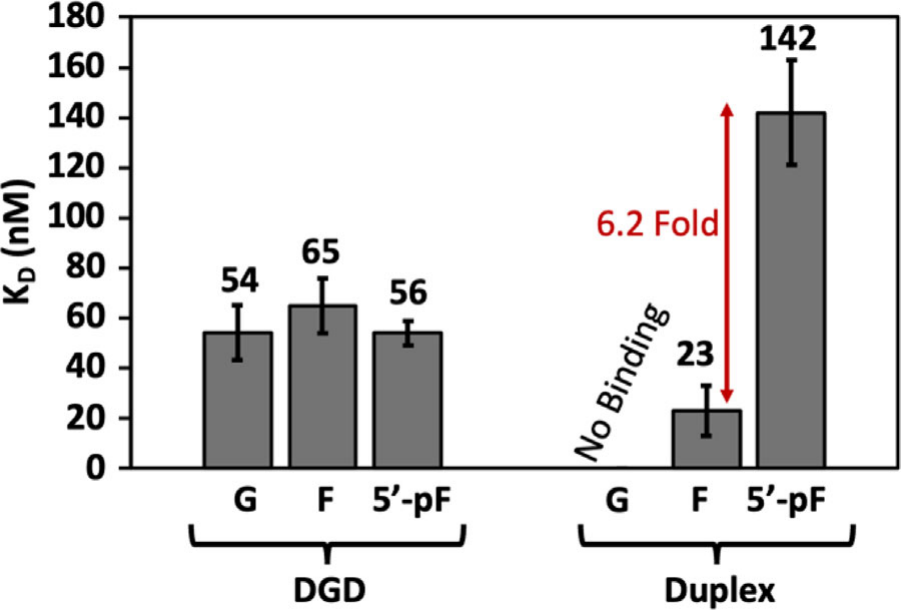
The *K*_D_ values for the D210A‐APE1 DNA binary complexes measured by fluorescence anisotropy for the central loop modified DGD and duplex motifs. Studies performed on substrates containing dG, F, or 5’‐pF with catalytically inactive APE1 mutant D210A. These data are adapted from a previous report from our laboratory [37].

*D210A‐APE1 Binding Affinity to DGD Scaffold‐* Binding studies were conducted by fluorescence anisotropy to obtain *K*_D_ values for the binding interaction between D210A‐APE1, an active‐site mutant that permits binding but not cleavage of most substrates, and the central loop‐modified DGD in comparison to duplex, a highly favored APE1 substrate. Having dG present at this position is indicative of a nonmodified system. Substituting this dG with F transforms it into an APE1 cleavage substrate, which upon reaction would produce 5’‐pF (Figure 1). In the duplex context, APE1 will not bind when the unmodified dG is present. When F is present in the duplex context, D210A‐APE1 tightly binds with a low average *K*_D_ value of 23 nM, but when the product 5’‐pF is present, binding is weakened by 6.2‐fold with a measured average *K*_D_ value of 142 nM (Figure 7). In contrast, D210A‐APE1 could bind to all studied variations of the central loop DGD with about the same affinity. Within error, the average *K*_D_ values ranged from 54–69 nM (Figure 7). This means APE1 can bind to its cleavage product, 5’‐pF, just as tightly as its substrate, F, in the central loop DGD scaffold. Surprisingly, APE1 also binds to the DGD with no F present; that is, a G4 motif lacking an AP site or its analog is sufficient for APE1 binding.

## 3 Discussion

These studies began with the hypothesis that thermodynamically folded G4 structures are perhaps a mixture of folded, noncleavable G4s, and other structures that are neither a G4 nor a classic single‐stranded structure (Figures 2A, B) and that these non‐G4 structures are substrates for APE1 enzyme activity. Thus, the cleavage yield represents the fraction of the sequences adopting the non‐G4 and nonsingle‐stranded structure. The discussion that follows provides support for this hypothesis.

Herein, endonuclease cleavage activity for APE1 targeting F‐modified *VEGF* DGD scaffolds was explored. Structural inspection by CD analysis (Figure 3) revealed that placing F in the loop positions allowed G4 formation in both the ssG4 and DGD scaffolds; however, CD does not report on the fraction of the sequence that actually adopted a G4, especially in the DGD context in which the spectra are dominated by the duplex handles. Alternatively, placing F in the core position inhibited G4 formation based on the CD analysis. These studies were performed in both K^+^, which enables G4 folding, and Li^+^, which does not [39]. The different ions do not impact the spectra of the non‐G4 scaffolds studied.

Additional studies were performed on a modified *VEGF* NMR strand with the PQS portion replaced with an F‐containing poly‐T segment opposite to the DGD poly‐T complement. This control mimics an unstructured region embedded between the duplex handles. The CD data for this poly‐T *VEGF* double‐strand construct in K^+^ aligns well with that of the core F DGD in K^+^, confirming the lack of G4 presence between the duplex handles in this DGD (Figure S3). Interestingly, the CD spectrum for the single‐strand non‐PQS (the (CAT)_8_ sequence, which has no secondary structure) and the CD spectrum for the *VEGF* NMR ssG4 in Li^+^ that is agnostic to G4 folding do not align and have different shapes (Figure S7). This signifies that the *VEGF* NMR ssG4 in Li^+^ is not a single‐stranded structure. In comparison to the literature, the CD spectrum for the *VEGF* NMR ssG4 in Li^+^ is similar to that of a spectrum reported by Liu et al., in which they studied a nonmodified *VEGF* sequence similar to our work in Cs^+^ solution, another cation agnostic to G4 folding [42]. Their work found this sequence adopts a structure that is neither single‐stranded nor a G4 but rather a “G‐coil” found with G‐rich strands. To our understanding, this can be an ensemble of noncanonical structures in G‐rich sequences, as well as an actual coil structure. Based on our ^1^H‐NMR studies for this sequence in Li^+^, the structure is dynamic and fails to give observable base pair imino signatures [37]. It is this short‐lived, dynamic structure with an F modification that we propose APE1 can cleave, not the G4 fold. To further explore this idea, we analyzed possible secondary structures using UnaFold software [https://doi.org/10.1007/978‐1‐60327‐429‐6_1]. This program will not predict G4 folds but will predict other noncanonical structures. Data on the *VEGF* NMR WT strand found strong potential for a variety of hairpin‐based structures with different lengths of duplex and different numbers of bubbles (Figure S8). Therefore, our ssG4 systems likely adopt various hairpin folds, but to a lower extent than G4 folds. The isolated PQS portion of the *VEGF* NMR WT sequence was also analyzed by UnaFold, as this segment has the potential to fold in the DGD systems. Data for this also depicted potential for adopting various hairpin folds (Figure S9). Therefore, we propose APE1 cleaves F‐containing noncanonical hairpin structures formed by PQS systems.

Initial support for the claim that APE1 is cleaving non‐G4 structures in PQS‐based reactions came from the assay using a gradient of Li^+^ and K^+^ ions to shift the equilibrium between G4 folded and non‐G4 structures while monitoring the APE1 activity (Figure 5). First, any sequence for which the structure based on CD did not change between K^+^ and Li^+^ salts (i.e., duplex, ss‐non‐PQS, and core F in either the DGD or ssG4) was found to give a consistent yield across the Li^+^/K^+^ salt gradient (Figure 5). The most striking observation is that the core F sequence in the ssG4 context consistently gave APE1 final cleavage yields similar to the preferred duplex substrate (∼90%; Figure 5), albeit with slower kinetics (Figure 4). If this G‐rich sequence adopted a single strand like the ss‐non‐PQS, the yield would be ∼5% instead of ∼90% at the same time point. Further, the cleavage yield stayed consistent, as there was no change in the structure across the salt gradient for these substrates. It is important to note that hairpin stability does not change in the presence of K^+^ vs. Li^+^, therefore there would be no change across this gradient if a hairpin is being cleaved [43]. This suggests that the non‐G4 structure that is formed is not a partial quadruplex, depending on K^+^, but rather duplex or hairpin‐like. On the other hand, both the central loop and 3’‐loop F DGD and ssG4 scaffolds showed the highest APE1 cleavage yield in the presence of only Li^+^ ions, and the yield decreased with increasing amounts of K^+^ ions, which drove the formation of G4 folds for these sequences. This supports the hypothesis that APE1 does not target F‐containing G4 motifs for cleavage.

The main difference we see between the ssG4 and DGD scaffolds in this study is the amount of APE1 cleavage that takes place in the Li^+^ dominant studies. When the G4 cannot form in the single‐stranded system, the cleavage of F by APE1 gave yields comparable to duplex at >90% (Figure 4A). In this situation, all three ssG4 scaffolds are non‐G4 and nonsingle‐stranded structures which APE1 prefers to cleave, likely dominated by the hairpin structures predicted by UnaFold (Figure S8). In contrast, in the DGD scaffolds, there is a strong APE1 cleavage yield dependency for the F modification location in Li^+^. As the modification location is positioned further from the 3’‐duplex handle, the yield decreased. These results align with previous studies by Hoitsma et al., who studied APE1 cleavage of an F in a primer template junction (PTJ) [6]. In the PTJ studies, the authors found that APE1 cleavage of an F, located in an unstructured region of DNA adjacent to duplex, was greatest when the F was closest to a 3`‐duplex and the yield decreased as the F was positioned further from the duplex. This is similar to our observation for F in the Li^+^ only and unfolded context, in which the 3`‐loop F closest to the 3`‐duplex gave the highest yield, followed by the core F, and then the 5`‐F loop. These studies identify that the duplex handles impact the structure and APE1 cleavage yield (Figure 4B).

In summary, the F modification located in non‐G4‐containing PQS scaffolds affects the cleavage yield by APE1 when it is in the DGD context, but not the ssG4 context. This is relevant to the decreased yield in the core F DGD when compared to the ssG4. Additionally, we can envision that another non‐G4 secondary structure is present, such as a G‐hairpin, that serves as a substrate for APE1 [42].

Activity assays were also performed over a time course to monitor the maximum cleavage yields of these DGD systems by APE1 in K^+^ solutions (Figure 4). The reactions were performed at a 1:1 ratio of DNA and APE1. As expected, APE1 quickly cleaved F in a duplex context with >90% yield. In contrast, the single‐strand non‐PQS substrate was very slow to react and only reached 9% cleavage yield at 5 min, at which all other substrates had reached near completion of cleavage. Despite being slow to react, this substrate reaches a yield of 75% by 3 h (Figure S4). Interestingly, the core F ssG4 scaffold, which does not adopt a G4 fold, showed APE1 cleavage yield similar to that of the duplex context. Again, this highlights that the unfolded ssG4 is not the same as a canonical single strand, as it shows incredibly different cleavage yield by APE1. On the other hand, the F‐modified loop ssG4 scaffolds, which fold into G4 motifs, showed significantly lower final APE1 cleavage yields (Figure 4). The central loop ssG4 was cleaved at a final average yield of 17%, and the 3’‐loop gave a final average yield of 36%. The difference in yield between loop F ssG4 scaffolds could be due to locational dependency and/or differences in loop length. The time course APE1 cleavage assays on the DGD systems found that the yields for the F‐modified loop DGD scaffolds stayed about the same as the ssG4 scaffolds, but the core F DGD had a significantly lower yield, 53%, compared to the ssG4. This brings into question the different types of structures that could be occurring across the different scaffolds.

To test the hypothesis that the ssG4 sequences could be folding into hairpin motifs that are cleaved by APE1, we performed this time reaction assay on a hairpin structure with a similar sequence to the *VEGF* NMR sequence (Figure 2). CD studies were performed on the hairpin to show the lack of a G4 (Figure S10). These studies found that APE1 can cleave this F‐containing hairpin at around 66% at 5 min and found similarity to the core F motifs in the way it was slower to react compared to the G4 forming motifs (Figure S6). The fact that this hairpin can be cleaved efficiently by APE1 and exhibits similar trends as the non‐G4 forming motifs, in terms of the time curve, supports the hypothesis that APE1 could be cleaving hairpins in the PQS‐based reactions. As already identified by UnaFold data, the structure of hairpins in our *VEGF* G4 motifs can vary dramatically, which likely can impact the cleavage yield percentage by APE1. Additionally, APE1 endonuclease activity on the poly‐T double‐stranded system with an unstructured region between duplex handles was studied over the time course. Take note from the PTJ studies that F location can impact this yield [6]. Interestingly, the poly‐T double‐strand reached 60% at 5 min, and the hairpin time curves nearly aligned (Figure S6). This suggests that hairpins and unstructured single‐stranded regions adjacent to duplex containing F‐modifications can both be cleaved in a similarly efficient manner by APE1. As explained, these cleavage yields can likely vary in both motifs, and this data is not indicative of these types of motifs consistently being equally good substrates for APE1. Additionally, the poly‐T double‐strand type motif could only be present in DGD motifs, but the hairpin could form in either ssG4 or be embedded between duplexes in DGD motifs. In conclusion, if either of these structures is present during PQS reactions, it will likely be cleaved by APE1. Although, it is more likely hairpin structures being cleaved in these PQS‐based assays.

Then, still curious about other contributing factors to low yields in G4 systems, we moved on to product inhibition‐based studies. These activity assays were performed with APE1 and the DGD scaffolds compared to duplex substrates (Figure 6). For these studies, APE1 was added in approximately 0.2 equivalents over 5 additions to approximately 1 equivalent of DNA with 5‐minute intervals in between. This gives ample time for APE1 turnover in between additions in addition to introducing more APE1 in case the present APE1 is inhibited. Interestingly, this data can simultaneously give insight into whether there are uncleavable substrates in solution. The duplex substrate reached its maximum yield of >90% after 5 min of the first 0.2 APE1 addition and remained at the same yield with the remaining additions. Additionally, both loop F‐modified DGD scaffolds showed minimal increase from the first to the last APE1 addition, showing similar low yields as observed in previous time and Li^+^/K^+^ studies. In this case, this data gives less information about product inhibition and more about cleavable substrates in solution. Reaching their maximum yields, according to other data from these studies, from the first APE1 addition and remaining at that yield with additional APE1 additions, suggests that there is nothing left that can be cleaved. For duplex, this is likely a portion of improperly folded DNA that cannot be cleaved. On the other hand, this once again supports the idea that APE1 cannot cleave the largest population in these loop F DGD solutions, the G4‐based DGD scaffolds.

In contrast, core F DGD experienced far larger increments of increasing yields with each APE1 addition. The first APE1 addition yielded 18% cleavage and continuously increased until the final addition yielded 59%. As previously mentioned, the location of the F site decreases the yield in this situation, which would explain why the yield never exceeds previous yields observed in these studies. These results are indicative of product inhibition, as more APE1 is needed to increase yield each round, instead of the already present APE1 turning over product to move on to the next substrate. This tells us that the poorly folded DGD, similar to a PTJ structure, does cause APE1 product inhibition.

With questions still remaining about the impact of product inhibition on the loop F DGD scaffolds, we came back to previously obtained binding data for the central loop F DGD [37]. The dissociation constants for the catalytically inactive mutant D210A‐APE1 bound to the central loop F DGD substrate and cleavage product binary complexes were measured by fluorescence anisotropy to reveal structure‐dependent binding differences in comparison to duplex (Figure 7). Consistent with the literature, APE1 tightly binds F‐modified duplex and shows sixfold weaker binding to the cleavage product [8, 25]. On the other hand, this binding data shows that APE1 binds the central loop G, F, and 5’‐pF DGD scaffolds with similar affinity. The question arises, which structure is APE1 binding with the reported affinity, the folded G4 or the non‐folded state? This question would need to be addressed by single‐molecule binding methods [44].

The present studies indicate APE1 cleaves F‐containing non‐G4 structures, those likely being hairpin motifs. Future in‐cell studies will build on our previously validated reporter assay framework to determine whether the structural context that modulates APE1 cleavage in vitro also produces distinct transcriptional or repair outcomes in a cellular environment [15, 17]. Incorporation of recently developed DGD‐based plasmids will enable assessment of the impacts of these structures in a physiologically relevant environment [45].

## 4 Conclusions

In these studies, we aimed to monitor APE1 cleavage of F, an abasic site mimic, in a DGD model genomic context. The studies revealed that APE1 cleaves the fraction of the population that fails to adopt a G4 fold. Support for this proposal includes the CD data showing a unique, noncanonical structure when F‐containing ssG4 is in Li^+^ solution, which aligns with CD data from the literature that found this sequence can adopt a non‐G4 structure that the authors refer to as a G‐coil [42]; we propose that this non‐G4 structure may be duplex or “hairpin‐like.” The APE1 activity assays identified efficient cleavage yields similar to duplex cleavage. These assays also revealed that increasing the presence of a folded G4 results in decreasing APE1 endonuclease activity by using a mixture of Li^+^ and K^+^ to control the level of G4 folding (Figure 5). This supports the idea that APE1 does not cleave well‐folded G4 scaffolds, even if they contain an AP(F). Rather, APE1 appears to be cleaving alternative, non‐G4 structures present to varying degrees in the DGD scaffolds. These alternative structures are not simple single‐stranded ones, based on CD, but are also not fully folded G4s. They may be G‐hairpins; this question remains to be addressed by structural methods. The present work highlights that DNA secondary structure controls APE1 endonuclease activity, with G4 motifs showing inhibitory effects. Questions remain regarding APE1 cleavage of an AP in the genomic context in G‐rich sequences that can adopt G4 structures; for example, does cleavage of the non‐G4 folded population provide the strand break that then allows rapid R‐loop and/or G4 formation to occur? [37, 46]

## 5 Methods

*APE1 activity assays‐* Using the purified and annealed samples described in the supporting information, individual reactions were run in 25‐µL samples of 100 nM nonradiolabeled DNA plus 14–18 nM radiolabeled DNA. The DNA‐containing samples were pre‐incubated at 30°C for 5 min. 30°C is below the low *T*_m_ values of the duplex handles of the DGD scaffold, and reactions at this temperature will minimize destabilization [37]. Immediately before incubation, 1 mM DTT and 1 mM Mg(OAc)_2_ were added to each reaction sample. Once the samples were adjusted to the reaction temperatures, the appropriate volume of APE1 was added to achieve a concentration of 100 nM, and the reaction sample was returned to 30°C. At the desired time point, the reaction samples were removed from the heat block and quenched by adding 5 µL of a 6x stop buffer comprised of 100 mM EDTA, 50% glycerol, and 0.25% w/v of the xylene cyanol and bromophenol blue dyes. For the time course activity assay, the individual reactions were quenched at 30 s, 1 min, 2 min, 5 min, 10 min, and 15 min. For all other activity assays, the reactions were quenched at 5 min, including product inhibition studies, which introduced 0.2 equivalents of APE1 every 5 min, taking an aliquot 5 min after each addition and quenching it. Negative controls were run in the absence of APE1. Quenching with the stop buffer was immediately followed by denaturing the reaction samples at 65°C for 20 min. The samples were further denatured at 90°C for 5 min right before moving onto the next step. Upon this final denaturation, the samples were loaded into the 20% 7 M urea‐denaturing PAGE gel, which was followed by electrophoresis at 75 W for 2 h. The gels were placed under a storage phosphorimager screen, exposed for 18 h, and then the bands were visualized by autoradiography (TyphoonTM 9400 Variable Mode Imager (GE Amersham Biosciences)) and quantified using ImageQuant image analysis software. The percent cleavage yields of each reaction were calculated by dividing the band intensity of the product band by the sum of the intensities of the product and reactant bands and then multiplying by 100. All reactions were run in triplicate to calculate standard deviation and provide error bars for the data. Graphs and figures were constructed using Microsoft Excel, Origin, and BioRender.

## Conflicts of Interest

The authors have no conflicts of interest to report.

## Supporting information



Supplementary data are available.
