# Supplementary material for: APE1 Activity is Controlled by Non‐G‐Quadruplex Conformations in Single‐ and Double‐Stranded G‐Quadruplex Constructs
Source: Chemistry. 2025 Dec 17;32(5):e03023. doi: 10.1002/chem.202503023 (PMC12865141; doi:10.1002/chem.202503023)
Supplement: Supplementary file 1 — Supplementary data are available. [file CHEM-32-e03023-s001.pdf]

APE1 Activity is Controlled by Non-G-Quadruplex Conformations in Single- and Double-Stranded G-Quadruplex Constructs

Brianna L. Trabucco, Aaron M. Fleming and Cynthia J. Burrows*

Department of Chemistry, University of Utah, 315 S 1400 E, Salt Lake City, UT 84112-0850

*To whom correspondence should be addressed: burrows@chem.utah.edu

<u>**Item**</u>	<u>**Page**</u>
**Methods**	S2
**Figure S1**. CD data for duplex, *VEGF* NMR ssG4 and DGD	S4
**Figure S2**. Native PAGE gel for duplex, DGD and ssG4.	S5
**Figure S3.** Comparison of CD data for non-canonical double-strand systems in K+	S6
**Figure S4**. [Mg^{2+}] activity assays	S7
**Figure S5**. Time-based activity assay for single-strand non-PQS	S8
**Figure S6.** Time-based activity assay for Poly-T Double-Strand and Hairpin	S9
**Figure S7**. Comparison of CD data between single-strand substrates in Li+	S10
**Figure S8**. UnaFold DNA folding predictions for VEGF NMR WT Strand	S11
**Figure S9.** UnaFold DNA folding predictions for VEGF NMR WT PQS	S13
**Figure S10**. CD data for Hairpin	S15
**References**	S16

Methods

DNA and Protein Preparation- Purification and oligomer stock concentration purities were previously reported by our laboratory.[37] The WT APE1 used for activity assays and catalytically inactive D210A mutated APE1 proteins for binding studies were prepared as previously reported by our laboratory.[8] Pure dG-rich oligomers were 5'-^{32}P radiolabeled using γ-^{32}P-ATP and T4 polynucleotide kinase. The crude radiolabeled G-rich strands were purified using a PD Spin-TrapTM G-25 column (Cytiva).

A thermodynamic folding protocol was used to obtain the DGD scaffolds. First, solutions of 100 nM dG-rich non-radiolabeled strand and approximately 10% excess 5'-^{32}P radiolabeled strand in a buffer mixture of 20 mM Tris (pH 7.4) and 100 mM KOAc or LiOAc were denatured by heating to 90 °C in a hot bath for 5 minutes. Once denatured, the hot bath with the denatured sample was turned off and brought back to room temperature over a ~3 h period. For double-stranded scaffolds, the complementary strand (1.2-fold excess to G-rich strand) was added to the solution once it reached room temperature. The room temperature sample was then placed at 4 °C for 24 h to complete the annealing process.

Conformational states of the annealed DNA samples were monitored by native polyacrylamide gel electrophoresis (PAGE) under non-denaturing conditions using a 12% acrylamide gel with both radiolabeled and non-radiolabeled DNA present in the buffer system mentioned above. The gels were run at 10 W for 5-7 h at 4 °C and placed under a storage phosphorimager screen to expose for 18 h; the bands were then visualized by autoradiography (TyphoonTM 9400 Variable Mode Imager (GE Amersham Biosciences)).

Circular Dichroism (CD) Analysis- For the CD analysis, 5 μM of the annealed DNA samples in the buffer system comprised of 20 mM Tris (pH 7.4) and 100 mM KOAc or LiOAc were placed in a 1-mm quartz cuvette at 20 °C. The CD spectra were monitored from 220-350

nm utilizing a Jasco J815 system. Buffer background was subtracted from the spectra for each DNA strand. The spectra provided ellipticity values that were converted to molar ellipticity values ([θ]) to create a plot consisting of [θ] on the y-axis and wavelength (nm) on the x-axis.

Fluorescence anisotropy binding assays- Fluorescence anisotropy experiments were performed by titrating the prepared 5'-FAM-labeled DNA samples with varying concentrations of the catalytically inactive D210A mutated APE1 protein from 0-5000 nM. Prior to analysis, the sample plate was incubated at 22 °C for 30 min. A BioTek Synergy2 Multi-Mode Microplate Reader was used to measure the excitation and emission wavelengths 485 and 520 nm which were utilized in calculating the K_D value between DNA and D210A-APE1 binding, according to our previous method.[37] Triplicate trials were performed to obtain the standard deviations of each data point and incorporate error bars into the plot.

Figure S1. CD data for duplex, *VEGF* NMR ssG4 and DGD

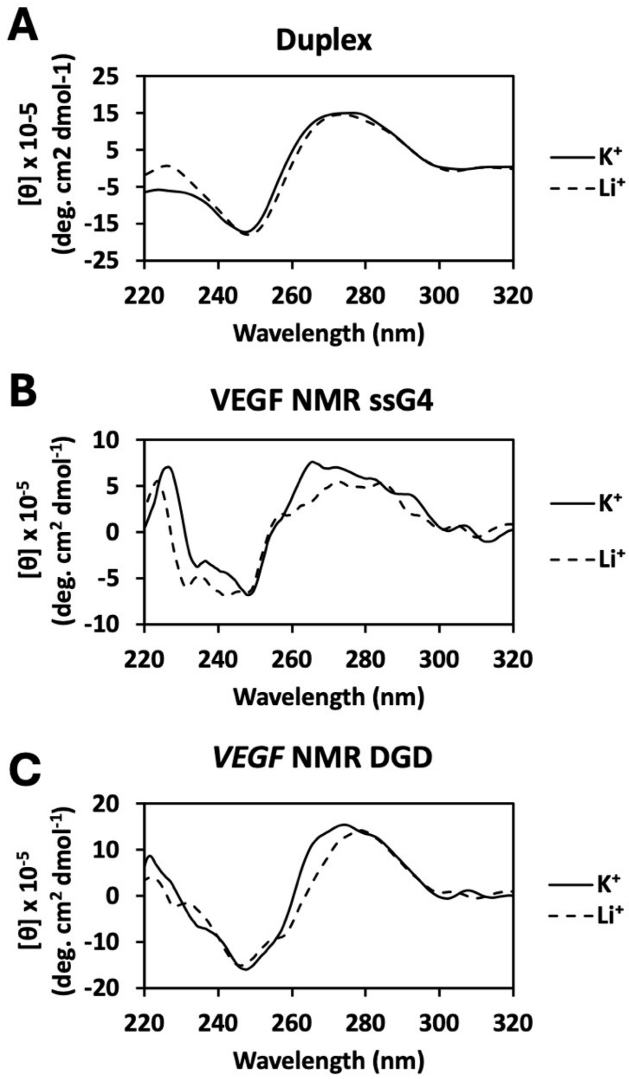

Additional CD experiments conducted with *VEGF* NMR sequence and F modified duplex

Figure S2. Native PAGE gel for duplex, ssG4, DGD and poly-T scaffolds.

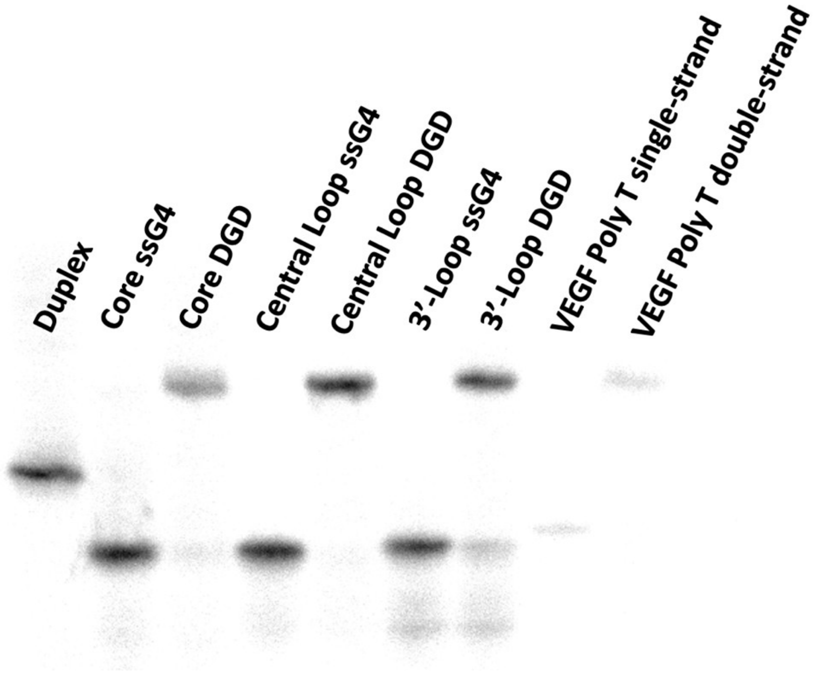

Native polyacrylamide gel electrophoresis (PAGE) using a 12% acrylamide gel with radiolabeled and non-radiolabeled DNA in K^+ buffer. Gels were run at 10 W for 5-7 h at 4 °C.

Poly-T *VEGF* Strand-

5'-CTATGTATACAAAGATTTTTTTTT**F**TTTTTTTTTTTAATGCGGCACGC-3'

Double-strand is annealed with poly-T complement.

Figure S3. Comparison of CD data for non-canonical double-strand systems in K^+

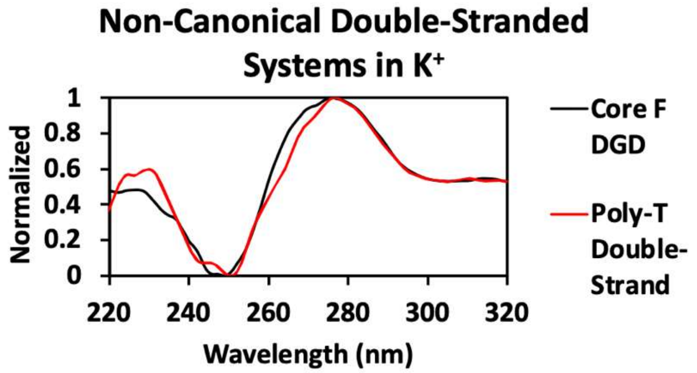

Additional CD experiments conducted with *VEGF* Poly-T Double-Strand and aligned with Core F DGD

Figure S4. [Mg^{2+}] activity assays

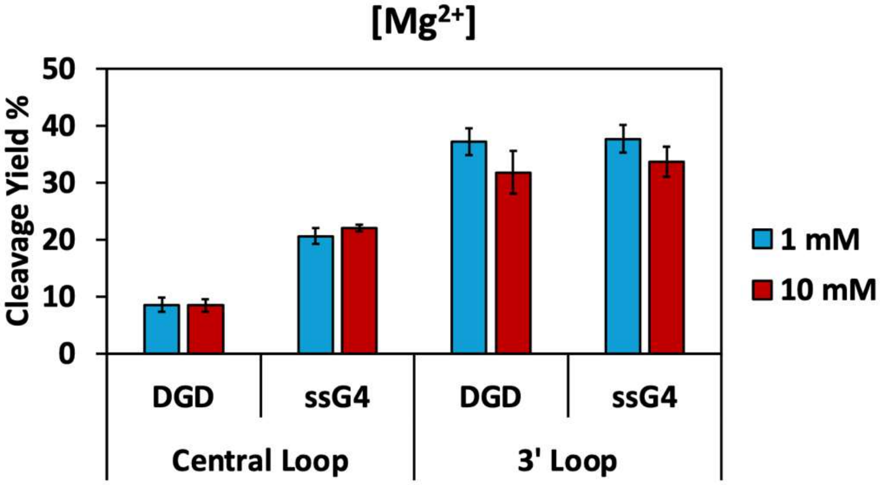

[Mg^{2+}] APE1 activity assays with loop F DGD and ssG4 scaffolds in 1 mM or 10 mM $Mg(OAc)_2$. Reactions were performed in solutions of approximately 100 nM DNA (5'-^{32}P radiolabeled and non-radiolabeled), 100 nM WT APE1, 20 mM Tris (pH 7.4 at 30 °C), 100 mM KOAc, 1 mM DTT and $Mg(OAc)_2$.

Figure S5. Time-based activity assay for single-strand non-PQS

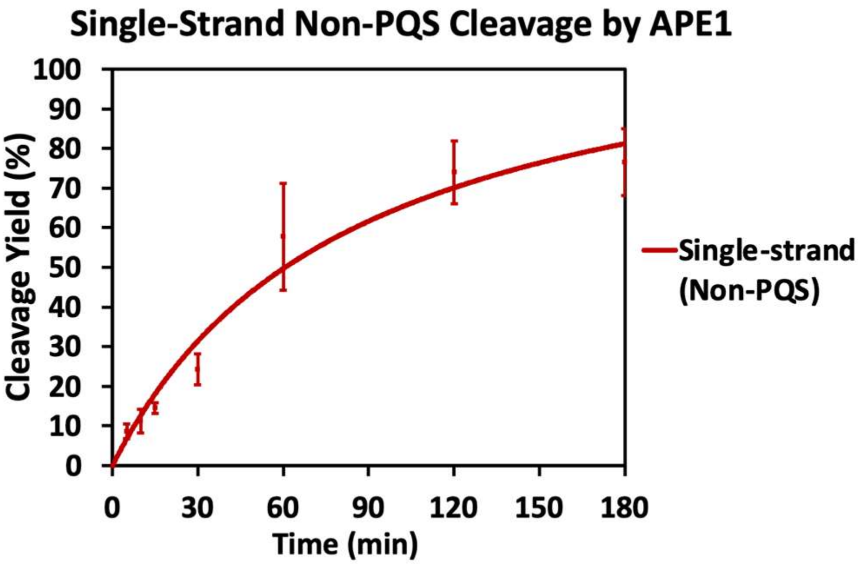

Time course APE1 activity assays with F modified single-strand non-PQS quenched at 30 s, 1 min, 2 min, 5 min, 10 min, 15 min, 30 min, 1 h, 2 h, 3 h. Cleavage yields under 5 min were undetectable. Reactions were performed in solutions of approximately 100 nM DNA (5'-^{32}P radiolabeled and non-radiolabeled), 100 nM WT APE1, 20 mM Tris (pH 7.4 at 30°C), 100 mM KOAc, 1 mM DTT and 1 mM $Mg(OAc)_2$.

Figure S6. Time-based activity assay for Poly-T Double-Strand and Hairpin

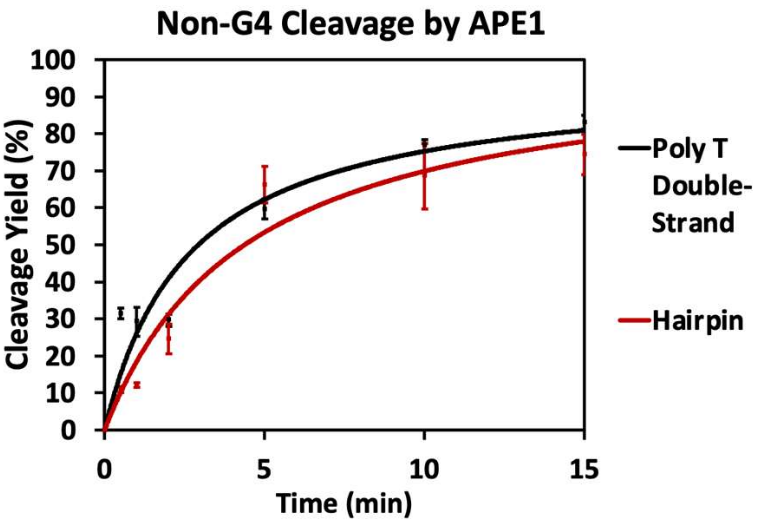

Time course APE1 activity assays with F modified Poly-T Double-Strand and Hairpin quenched at 30 s, 1 min, 2 min, 5 min, 10 min, 15 min. Reactions were performed in solutions of approximately 100 nM DNA (5'-^{32}P radiolabeled and non-radiolabeled), 100 nM WT APE1, 20 mM Tris (pH 7.4 at 30°C), 100 mM KOAc, 1 mM DTT and 1 mM $Mg(OAc)_2$.

Figure S7. Comparison of CD data between single-strand substrates in Li^+

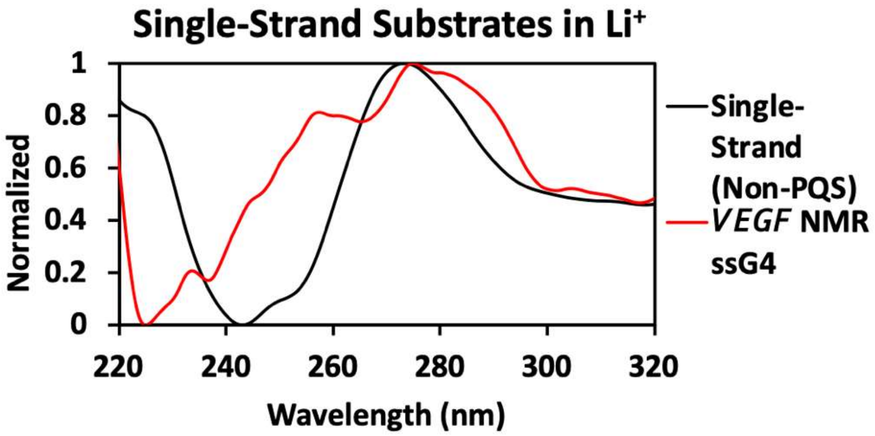

Additional CD experiments conducted with Single-Strand Non-PQS and aligned with *VEGF* NMR ssG4

Figure S8. UnaFold DNA folding predictions for *VEGF* NMR WT Strand

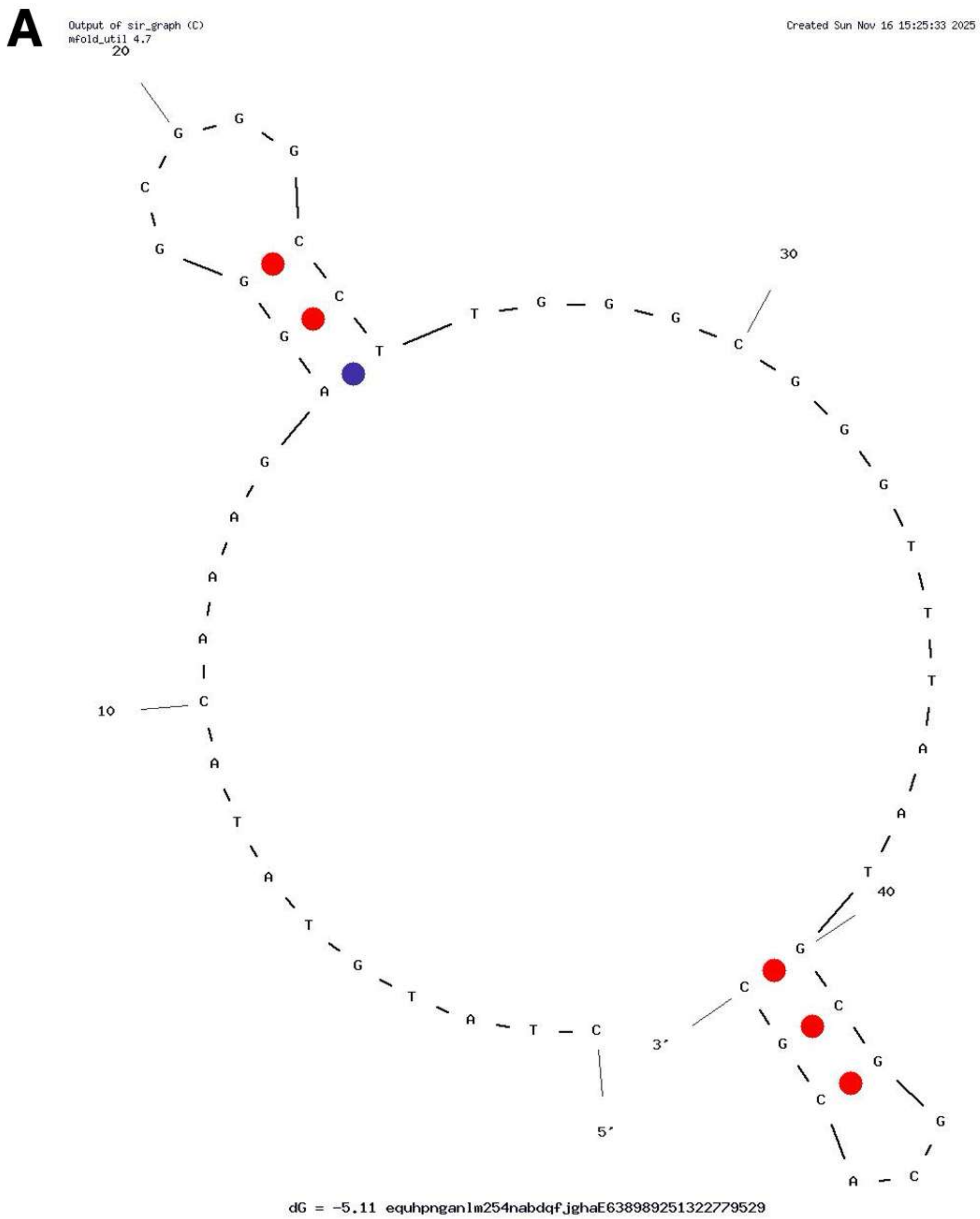

B

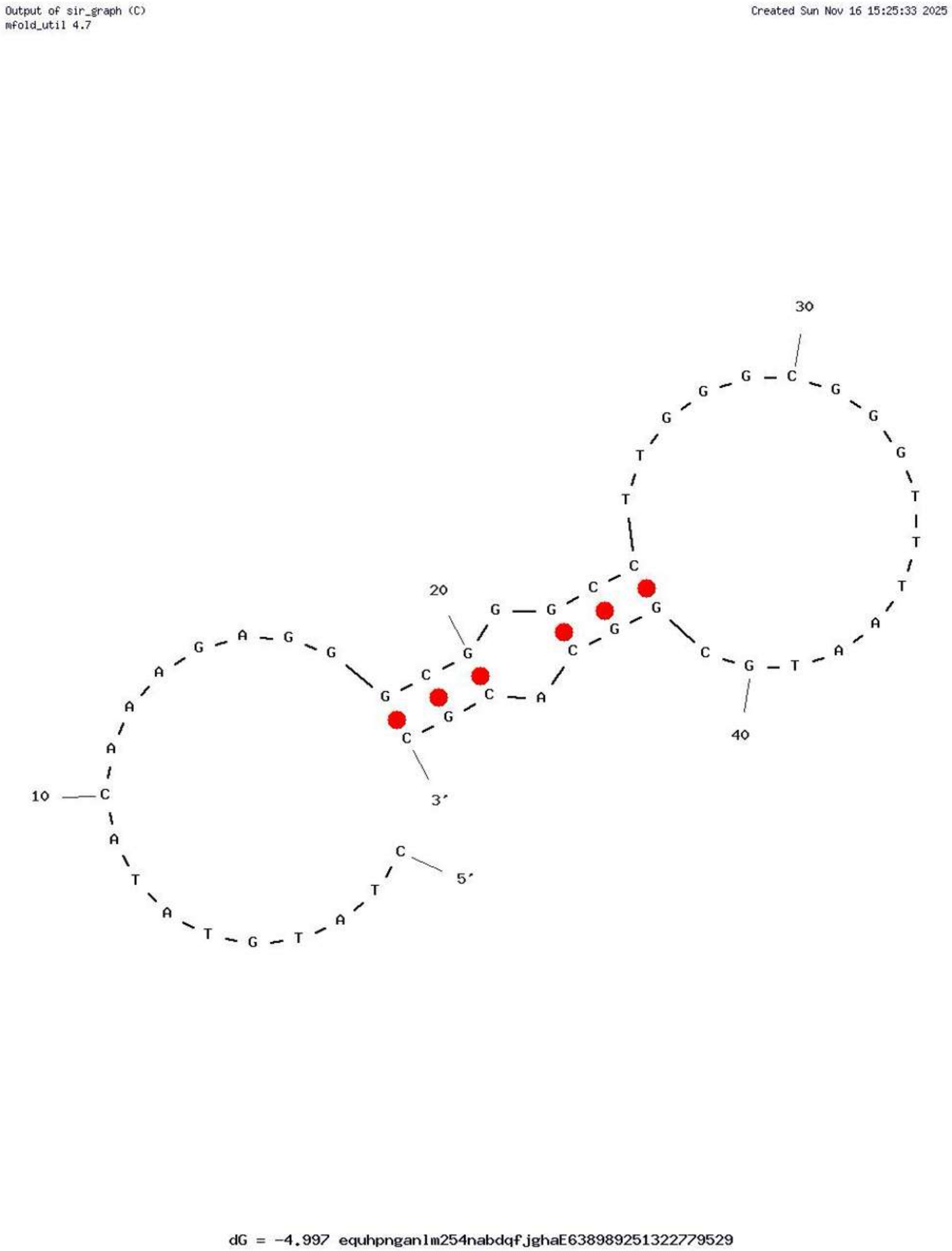
Output of sir_graph (C)
mfold_util 4.7
Created Sun Nov 16 15:25:33 2025
30
20
40
10
3'
5'
dG = -4.997 equhpnganlm254nabdqfjghaE638989251322779529

Figure S9. UnaFold DNA folding predictions for *VEGF* NMR WT PQS

A

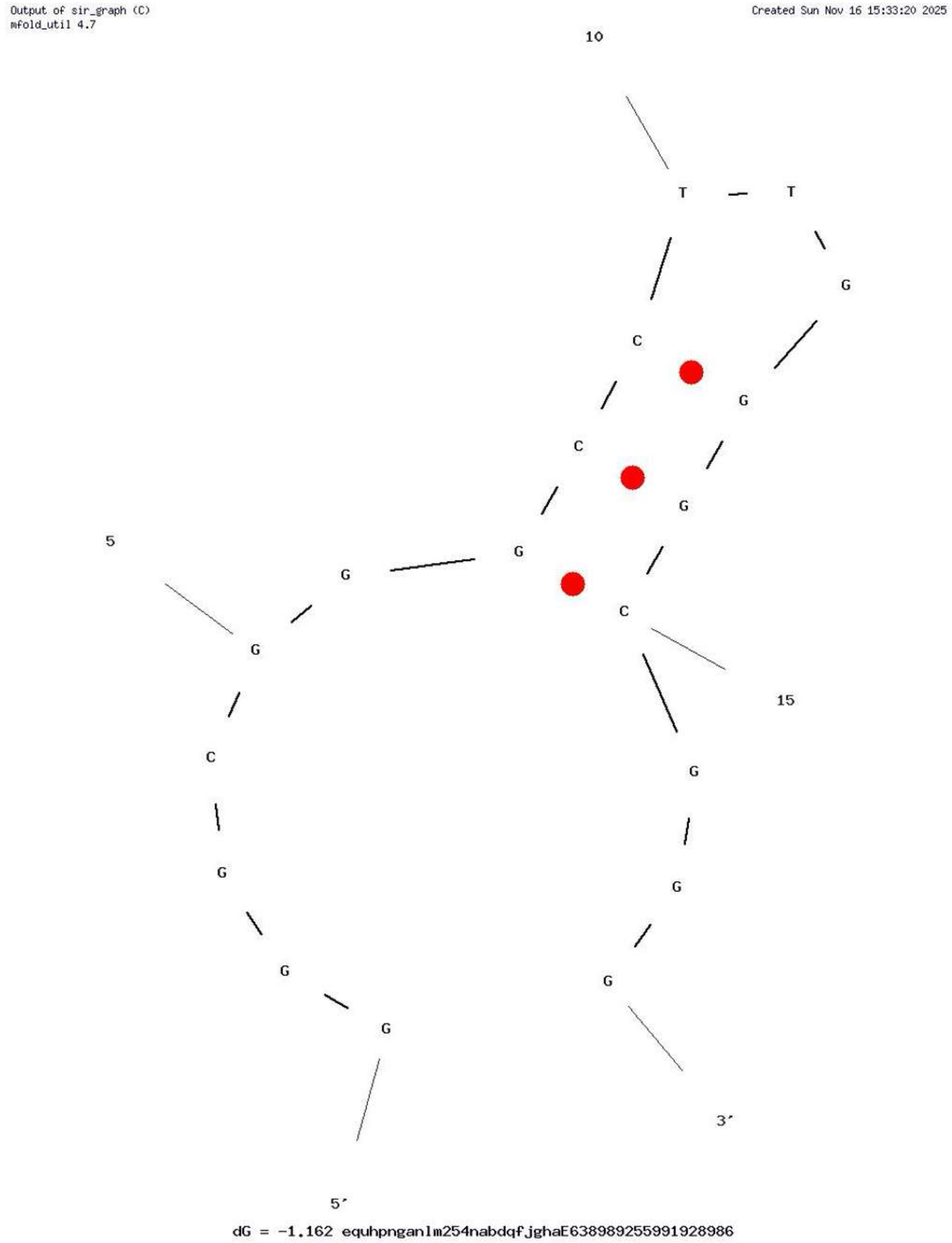

B

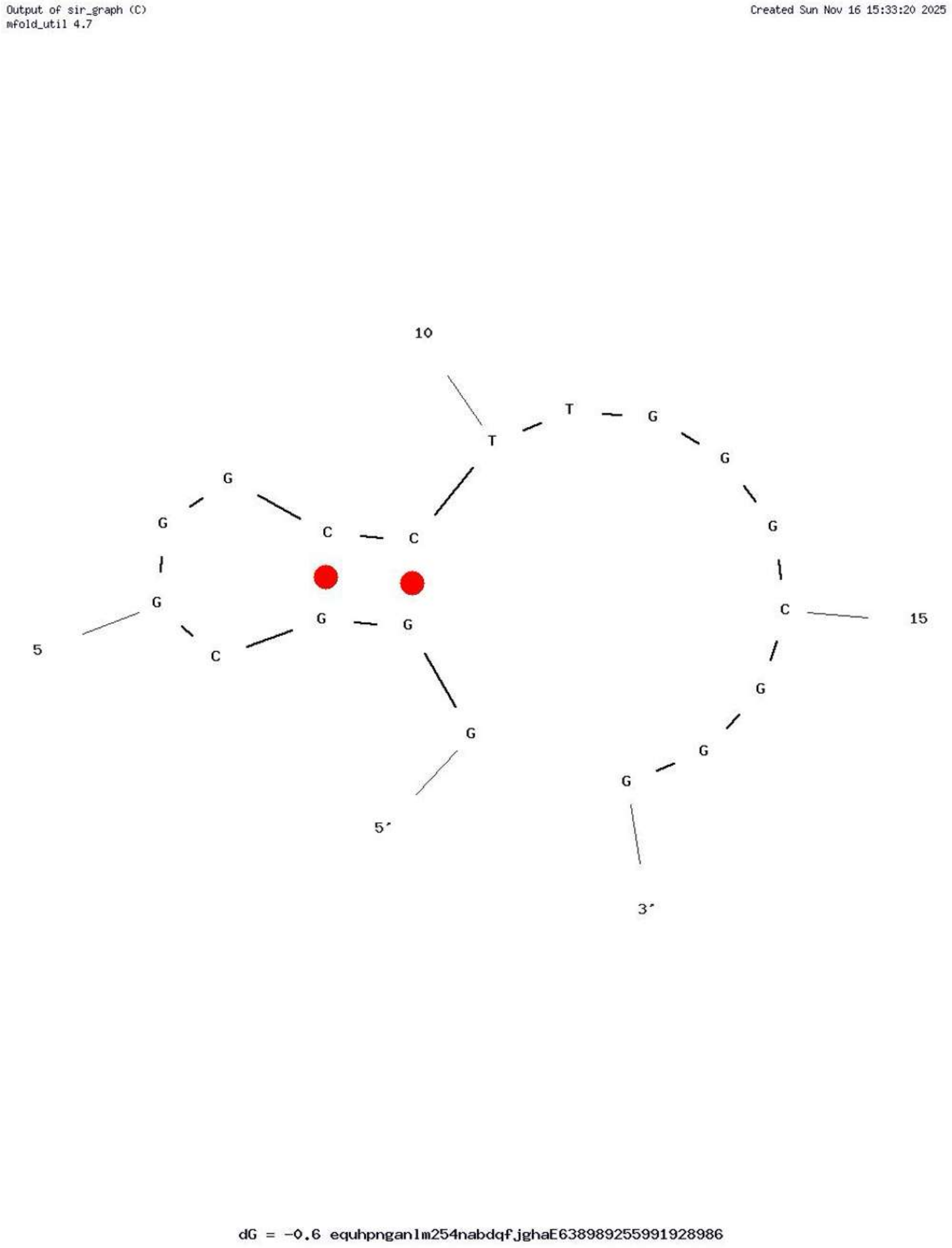
Output of sir_graph (C)
mfold_util 4.7
Created Sun Nov 16 15:33:20 2025
10
T
T
G
G
G
G
G
C
C
G
G
G
C
15
5
C
G
G
G
G
G
G
5′
3′
dG = −0.6 equhpnganlm254nabdqfjghaE638989255991928986

Figure S10. CD data for Hairpin

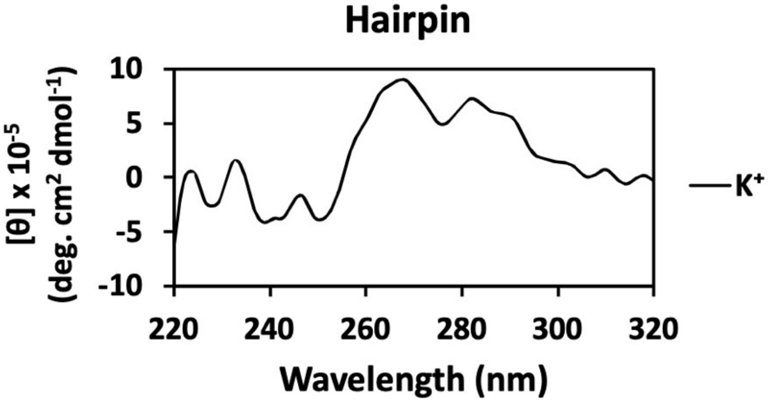

Additional CD experiments conducted with Hairpin control

Hairpin- 5'-GCAAAGAGGGCGGGCCTT**F**GGCGGGTTTGC-3'

The CD spectrum shows features characteristic of at least 3 different species in a mixture, including a G-triplex (~268 nm), single-stranded (~280 nm) and a hairpin (~285 nm).

References (from manuscript)

(8) Fleming, A. M.; Manage, S. A. H.; Burrows, C. J. Binding of AP endonuclease-1 to G-quadruplex DNA depends on the N-terminal domain, Mg(2+) and ionic strength. *ACS Bio. Med. Chem. Au* **2021**, *1* (1), 44–56.

(37) Fleming, A. M.; Guerra Castanaza Jenkins, B. L.; Buck, B. A.; Burrows, C. J. DNA damage accelerates G-quadruplex folding in a duplex-G-quadruplex-duplex context. *J. Am. Chem. Soc.* **2024**, *146* (16), 11364–11370.
